# Adult low-risk drinkers and abstainers are not the same

**DOI:** 10.1186/s12889-020-8147-5

**Published:** 2020-01-10

**Authors:** Janette Mugavin, Sarah MacLean, Robin Room, Sarah Callinan

**Affiliations:** 1 Centre for Alcohol Policy Research, School of Psychology and Public Health, La Trobe University, Bundoora, Victoria 3086 Australia; 2 School of Allied Health, Human Services and Sport, La Trobe University, Bundoora, Victoria 3086 Australia; 3 Centre for Social Research on Alcohol and Drugs, Department of Public Health Sciences, Stockholm University, Stockholm, 106 91 Sweden

**Keywords:** Adults, Low-risk drinking, Abstinence, Socio-demographics, Cross-sectional data

## Abstract

**Background:**

Alcohol consumption, even at low-levels, can not be guaranteed as safe or risk free. Specifically, the 2009 Australian National Health and Medical Research Council drinking guidelines recommend that adults should not drink more than two standard drinks on any day on average, and no more than four drinks on a single occasion. Nearly 40% of Australians aged 12 years and older drink alcohol but don’t exceed these recommended limits, yet adult low-risk drinkers have been largely overlooked in Australian alcohol survey research, where they are usually grouped with abstainers. This paper examines the socio-demographic profile of low-risk drinking adults (18+ years old), compared to those who abstain.

**Methods:**

Data from the 2013 National Drug Strategy Household Survey were used. In the past 12 months, 4796 Australians had not consumed alcohol and 8734 had consumed alcohol at low-risk levels, accounting for both average volume and episodic drinking (hereafter low-risk).

**Results:**

Multivariate logistic regression results indicated that low-risk drinkers were more likely to be older, married, Australian-born, and reside in a less disadvantaged neighbourhood compared with abstainers. There was no significant difference by sex between low-risk drinkers and abstainers.

**Conclusions:**

The socio-demographic profile of low-risk drinkers differed from that of abstainers. Combining low-risk drinkers and abstainers into a single group, which is often the practice in survey research, may mask important differences. The study may support improved targeting of health promotion initiatives that encourage low-risk drinkers not to increase consumption or, in view of increasing evidence that low-risk drinking is not risk free, to move towards abstinence.

## Background

The prevalence of alcohol consumption is high among Australians, with 75% of the population aged 12 years and older having consumed at least one Australian standard drink (10 g ethanol) in the previous year [1]. National sources also state that most adults drink at low to moderate levels [1, 2]. Low-risk drinking is defined two ways in the 2009 National Health and Medical Research Council (NHMRC) drinking guidelines: drinking no more than two Australian standard drinks per day, on average, to reduce the lifetime risk of alcohol-related chronic disease (i.e., lifetime low risk); and drinking no more than four standard drinks to reduce the risk of acute harms arising from a single drinking occasion (i.e., single occasion low risk). These guidelines are directed towards persons aged 18 years and older (i.e., of legal drinking age); younger Australians, particularly those aged 15 and under, are advised to abstain from alcohol [3].

Recent estimates suggest that 58% of Australians aged 12 years and over drink alcohol at lifetime low-risk levels, and on the measure of single occasion risk, 39% drink at low-risk levels. Accounting for both guidelines, 37% drink alcohol within recommended levels [4]. Low-risk drinking is common among older adults, and with Australia’s ageing population [5], understanding the profile of this group may become more important.

However, alcohol consumption, like many other modifiable behaviours, is not risk free, with recent studies suggesting that amounts as small as half a standard drink (i.e., 5 g) daily is associated with an increase in cancer risk [6]. Outside of morbidity and mortality work, few Australian studies differentiate between alcohol abstainers and low-risk drinkers or consider differences in demographic characteristics of the two groups. Even less is known about the attributes of low-risk drinkers who neither drink at levels associated with long-term nor short-term harm. Many Australian studies investigating the association between socio-demographic dimensions and alcohol use among adults have focused on elevated levels of drinking and the potential of long-term harm [7], short-term harm [8, 9], or both [10–13]. In such analyses, the exact configuration of the group to which risky drinkers are compared is often unclear [7, 8, 10, 12]. When information is provided, the comparison group typically includes both abstainers and non-risky drinkers [11].

Australian studies that differentiate between abstainers, low-risk drinkers and risky drinkers, and report socio-demographic correlates associated with alcohol use, tend to pool teenagers and adults together [14] or focus on a subset of adults based on sex [15, 16], age [17–20] or other characteristics (e.g., ethnicity) [21]. Consequently, socio-demographic information about Australian adults who drink alcohol, but do so at low levels, is limited.

Similarly, non-Australian studies have examined the socio-demographic correlates of abstainers, light or moderate drinkers and heavier drinkers, though they typically focus on discrete groups such as men [22] or an age cohort [23, 24]. Furthermore, direct comparisons between abstainers and low-risk drinkers is the exception rather than the rule [25]. Overall, these studies do provide some insights into social demographic differences between abstainers and low-risk drinkers. For example, a Finnish twin study found that former drinkers had a lower annual income, and they also spent less time per year in gainful employment over a 20-year period compared with moderate drinkers [26]. In this study, the measure of moderate drinking was comparable to the 2009 NHMRC long-term low-risk threshold. These studies examine attributes associated with one or more types of low-risk drinking but, as is the case with Australian-based studies, low-risk refers to either average total volume or episodic drinking and not to those who meet both criteria.

In this paper we address whether the socio-demographic characteristics of adult low-risk drinkers differ from those who did not consume alcohol in the past year. This is important in the current policy context as cultural-political assumptions in Australian thinking tend to favour the ideal of low-risk drinking as a goal. The long twentieth-century reaction against temperance [27] has meant that abstention from alcohol is still unexpected and even questionable in many social circles, while ‘low-risk drinking’ is the explicit ideal, for instance, in the Australian drinking guidelines [3], and the implicit ideal in such government campaigns as the National Binge Drinking Strategy of 2008–2012 [28]. But who the low-risk drinkers are, especially those who drink within both the NHMRC guidelines, and whether or not they have the same socio-demographic characteristics as abstainers remains unanswered by the current literature.

## Methods

### Sample

Data were taken from the 2013 National Drug Strategy Household Survey (NDSHS), a nationally representative, cross-sectional survey of Australians aged 12 years and older [29]. A multi-stage stratified sampling design was used to randomly select residential households from across Australia, and one resident (aged 12 and over) from each of the selected households was randomly chosen. A ‘drop and collect’ method was employed: selected residents were given a paper version of the survey to self-complete, and the survey was collected at a pre-arranged date. The final sample included 23,855 respondents (49.1% response rate). A detailed description of the method is reported elsewhere [30].

In the present analysis, respondents aged 17 or younger (*n* = 1159) were excluded, along with 823 cases with incomplete data for alcohol consumption, leaving a sample of 21,873 for analysis. The focus on adults (18+) is consistent with the age parameters attached to the lifetime low risk and single occasion low risk guidelines [3].

### Measures

#### Alcohol use and risk levels

Past year alcohol use was based on whether the respondent had consumed alcohol in the past 12 months (yes/no). Patterns of drinking were measured by the graduated-frequency method, which asks about the frequency of drinking eight different quantities (ranging down from 20+ drinks to none) in the past year. An annual total volume was calculated from the graduated-frequency responses [31] and amounts reported ranged from zero to 7665 drinks.

Respondents were classified as abstainers if they reported no alcohol use in the past year or had a total volume of zero (*n* = 4796). This paper does not differentiate between never drinkers and former drinkers.

Two measures were used to classify respondents who had consumed alcohol in the past year: lifetime low risk (LLR) and single occasion low risk (SOLR). These measures were aligned with the 2009 guidelines [3]. Respondents with a total volume of 1–730 drinks were classified as lifetime low-risk drinkers, which is consistent with the average of below two drinks per day (over a single year) interpretation of the LLR guideline [32]; 13,081 respondents met this.

SOLR equated to the second guideline - drinking no more than four drinks on any single occasion [3] in the past year; 9194 respondents met this. A total of 8734 respondents drank in accordance with both guidelines; this category is hereafter referred to as ‘low risk’. Respondents drinking in excess of the thresholds (i.e., total volume > 730, or 5+ drinks on a single occasion) were categorised as ‘at risk’ and excluded from the main analysis. As Table 1 shows, 22.6% of adult Australians had not consumed alcohol in the previous year, and 37.0% drink at low-risk levels, taking into account both average volume and episodic drinking. These two groups were used in the main analysis.

**Table 1:** Percentage prevalence of lifetime risk by single occasion risk as per 2009 NHMRC Guidelines, Australians aged 18+, weighted 2013 national survey

Lifetime risk	Abstainer	Low risk (< 5 drinks on a single occasion)	At risk (5+ drinks on a single occasion)	Total
Abstainer	22.6	–	–	22.6
Low risk (<=2 drinks per day, on average)	–	37.0	22.1	59.1
At risk (> 2 drinks per day, on average)	–	1.7	16.6	18.3
Total	22.6	38.7	38.7	100

Socio-demographic variables included sex, age (in age groups), country of birth, marital status, number of dependent children in the household aged 14 and under and children older than 14 who are not financially independent and whom the respondent is parent or guardian for, highest educational qualification, pre-tax annual household income from all sources, neighbourhood disadvantage and geographical location.

Neighbourhood disadvantage was based on multiple socio-economic indicators of a neighbourhood and is expressed as quintiles [33]. The first quintile equates to the 20% most disadvantaged neighbourhoods and the fifth quintile the 20% least disadvantaged neighbourhoods. Geographical location was formulated from the Australian Statistical Geography Standard for Remoteness Area structure whereby postcodes are used to classify areas as: major cities, inner regional, outer regional, remote and very remote [34]. The last three area types were collapsed in this paper.

### Statistical analysis

Logistic regression was used to examine the socio-demographic correlates of low-risk drinking compared to abstaining. Data were weighted to address any imbalances in the probability of a respondent being selected and to ensure that the data are as representative as possible of the general Australian population. Results from the bivariate and multivariate analyses are presented as odds ratios (OR) with 95% confidence intervals (95% CI). OR are based on weighted data; sample numbers are unweighted. Analyses were conducted using Stata version 14.0. Sex-specific models have not been reported, as preliminary analysis found few differences between men and women in socio-demographic prediction of low-risk drinking versus abstention.

## Results

Table 2 shows household income was a strong predictor of low-risk drinking compared to abstention, controlling for all other variables. Low-risk drinkers were significantly more likely to be in a high household income category compared to those with a low income. Residing in a neighbourhood with a lower level of disadvantage increased the likelihood of being a low-risk drinker, as did the attainment of a post-secondary school qualification. Older age (40–64 and 65+ years compared to 18–24 years) increased the likelihood of being a low-risk drinker, whereas widowed adults were more likely than those in a marriage-type relationship to abstain rather than drink at low-risk once other factors were controlled for. Being born in Australia was positively associated with low-risk drinking as was living in inner-regional area. No significant difference was found between sex and drinking status.

**Table 2:** Bivariate and multivariate logistic regression models predicting low-risk drinkers (8434) compared to abstainers (4796)

	Bivariate		Multivariate^a^	
	OR	95% CI	OR	95% CI
Sex				
Male	1 (Ref)		1 (Ref)	
Female	1.02	(0.93, 1.11)	1.07	(0.97, 1.19)
Age				
18–24	1 (Ref)		1 (Ref)	
25–39	1.46***	(1.19, 1.79)	1.20	(0.92, 1.57)
40–64	1.98***	(1.63, 2.40)	1.56***	(1.23, 2.07)
65 and older	1.35**	(1.11, 1.65)	1.58**	(1.18, 2.06)
Country of birth				
Australian	1.98***	(1.81, 2.16)	1.85***	(1.68, 2.05)
Other	1 (Ref)	1 (Ref)	1 (Ref)	1 (Ref)
Marital status				
Never married	0.68***	(0.59, 0.77)	0.86	(0.73, 1.02)
Widowed	0.54***	(0.46, 0.63)	0.67***	(0.55, 0.81)
Divorced/separated	0.83**	(0.72, 0.94)	0.95	(0.82, 1.10)
Married/defacto/life partner	1 (Ref)		1 (Ref)	
Dependent children in household^b^				
None	1 (Ref)		1 (Ref)	
One or more	1.12*	(1.02, 1.23)	0.93	(0.83, 1.04)
Highest qualification				
Yr 13 or equivalent or less	1 (Ref)		1 (Ref)	
Certificate or Diploma	1.56***	(1.41, 1.73)	1.43***	(1.28, 1.60)
Bachelor or higher	1.90***	(1.70, 2.12)	1.48***	(1.30, 1.69)
Household annual income				
Low ($51,999 or less)	1 (Ref)		1 (Ref)	
Middle ($52,000–$103,999)	1.84***	(1.63, 2.08)	1.68***	(1.46, 1.93)
High ($104,000+)	2.96***	(2.59, 3.39)	2.34***	(1.99, 2.74)
Neighbourhood disadvantage^c^				
1 (lowest)	1 (Ref)		1 (Ref)	
2	1.42***	(1.24, 1.62)	1.23**	(1.07, 1.42)
3	1.75***	(1.52, 2.00)	1.48***	(1.27, 1.71)
4	2.18***	(1.91, 2.49)	1.71***	(1.48, 1.98)
5 (highest)	2.67***	(2.32, 3.08)	2.19***	(1.86, 2.58)
Geographical location				
Metro	1 (Ref)		1 (Ref)	
Inner regional	1.29***	(1.15, 1.45)	1.29***	(1.13, 1.47)
Outer regional/remote	0.99	(0.87, 1.3)	1.11	(0.96, 1.29)

^a^ Odds ratios (ORs) were adjusted for all the variables listed in this table

^b^ Dependent children are children aged 0 to 14 and those aged over 14 who are not financially independent for whom the respondent is the parent or guardian of

^c^ Neighbourhood disadvantage equates to the Socio-Economic Index For Areas (SEIFA; high scores equate to low levels of disadvantage)

Outcome variable: low-risk (*n* = 8434) (1), abstainer (*n* = 4796) (0)

Ns are based on unweighted data and estimates (%, ORs and confidence intervals (CI) are based on weighted data. CI: *p* < .05 is *; *p* < .01 is **; *p* < .001 is ***

There were some differences between the bivariate and multivariate analyses. The following characteristics were significantly associated with low-risk drinking at the bivariate level, but not at the multivariate level: being 25–39 years old; never being married or being separated; and having more than one dependent child in the household. The differences were possibly due to an interaction between age and marital status.

## Discussion

This paper compares socio-demographic characteristics of Australian adults who drink at low-risk levels with abstainers. Multivariate findings indicate that drinking in accordance with the Australian drinking guidelines, as opposed to abstaining from alcohol, was associated with individual and community level characteristics. Specifically, being older, not widowed, Australian-born, having a higher income and higher-level education, residing in a less disadvantaged area and living in an inner regional area.

Our finding that higher socio-economic status increases the likelihood of low-risk drinking as opposed to abstaining is consistent with studies of older adults’ alcohol consumption [18, 23]. It suggests that further education and greater financial means are associated with a social position where drinking is potentially more affordable and where moderation is socially acceptable [35, 36].

Drinking behaviour is widely considered to be age-related, and often characterised by heavier episodic drinking during early adulthood, more frequent but lower overall consumption in mid-to-late adulthood [11, 37], followed by increased prevalence of abstinence in later life [23]. Recent studies have also shown that the transition to abstinence in later life is not as widespread as in previous generations [19]. Thus, our finding that older adults are more likely to report low-risk drinking than abstinence, after all other factors are controlled for, was not unexpected. It may also be the case that low-risk drinking is a norm violation for 18–24-year-olds – they are either heavier drinkers [38] or, as recent data suggest, abstaining from alcohol [1]. Widowed adults, as opposed to those with a partner, were more likely to abstain than consume alcohol at low-risk levels. This finding is supported by previous studies [25] and is consistent with the argument that marital-type relationships facilitate the consumption of alcohol [39, 40]. No significant difference was found by sex in drinking status in the current study at either the bivariate or the multivariate level. Given women are more likely to abstain, and they are also less likely to drink at risky levels [41], it’s not unexpected that no significant difference was found.

The study’s limitations stem from the cross-sectional data source which does not allow causal inferences to be drawn. Also, alcohol use was compiled from self-report items, including the standard graduated-frequency questions; all items referred to a 12-month recall window and respondents were asked to answer in terms of standard drinks, as this unit was explained to them. All these aspects have the potential to introduce recall measurement error, which may yield an underestimate of consumption [42–44] and thus presumably an overestimate of abstinence and low-risk drinking.

The response rate of 48.1%, although comparable to previous waves of the NDSHS [45], presents the potential of non-response bias. For example, a comparison of the 4179 respondents who returned a blank or unusable 2013 NDSH survey with those who returned a completed survey (23,855) revealed higher proportions of men and younger adults among the former [30]. Given the two attributes (male; younger) are commonly associated with heavy drinking patterns (e.g., [46]), it is possible that demographic differences between respondents and non-respondents may have biased alcohol estimates. There is also evidence to suggest abstainers may be over-represented amongst non-respondents [47].

Despite these limitations, gaining a better understanding of the social location of low-risk drinkers, as distinct from abstainers can be seen as a first step towards learning more about the social norms and stability of low-risk alcohol use. This is important in view of the increasing weight of evidence that low levels of alcohol consumption are not risk free. Furthermore, increasing our understanding of low-risk drinkers may provide additional ways to frame discussions around less harmful drinking patterns and promote this as an achievable and acceptable practice, especially if abstinence is not perceived as a viable outcome.

## Conclusion

At different points in Australia’s history, social and political directives have shaped low-risk and non-drinking aspirations [27], yet few studies have afforded actual practices in these areas much attention. By comparing low-risk drinkers with abstainers, this paper identifies important socio-demographic characteristics of both groups. Given the differences that emerged, we question the appropriateness of treating low-risk drinkers and abstainers as a single comparison group in non-mortality or morbidity studies investigating risky drinking. Differentiating the demographic characteristics of abstainers and low-risk drinkers here and in future studies will support targeted interventions.

## Data Availability

The Australian Institute of Health and Welfare manage the data collection and dissemination of the National Drug Strategy Household Survey and we are grateful to them for facilitating access to the data via the Australian Data Archive (ADA). The data was accessed through a formal application submitted to the ADA (https://ada.edu.au/accessing-data/).

## Abbreviations

ADA: Australian Data Archive
CI: Confidence intervals
LLR: Lifetime low risk
NDSHS: National Drug Strategy Household Survey
NHMRC: National Health and Medical Research Council
OR: Odds ratios
SEIFA: Socio-Economic Index For Areas
SOLR: Single occasion low risk
